# Improved heart hemodynamics after draining large-volume pleural effusion: a prospective cohort study

**DOI:** 10.1186/s12890-018-0625-5

**Published:** 2018-04-25

**Authors:** Zheng Wang, Qi-Zhe Cai, Cheng-Jun Ban, Duo Chen, Li-Li Xu, Xiao-Juan Wang, Zhen Wang, Yuan Yang, Xiu-Zhang Lv, Huan-Zhong Shi

**Affiliations:** 1 Department of Respiratory and Critical Care Medicine, Beijing Institute of Respiratory Medicine, Beijing Chaoyang Hospital, Capital Medical University, No. 8 Gongti Nanlu, Chaoyang District, Beijing, 100020 China; 2 Department of Echocardiography, Cardiovascular Diseases Research Institute, Beijing Chaoyang Hospital, Capital Medical University, No. 8 GongtiNanlu, Chaoyang District, Beijing, 100020 China

**Keywords:** Large-volume pleural effusion, Drainage, Hemodynamic, Transthoracic echocardiography

## Abstract

**Background:**

Pleural effusion (PE) drainage can relieve the symptoms of dyspnea; however, details of the resulting hemodynamic changes remain undefined.

**Methods:**

Subjects older than 12 years with massive PE requiring pleural drainage were included in this study. Hemodynamic parameters were collected using transthoracic echocardiography at pre-drainage, immediately post-drainage, and 24 h after drainage.

**Results:**

We enrolled 47subjects in this prospective study from June 9, 2015 to September 18, 2016 in Beijing Chaoyang Hospital and 28 subjects were analyzed finally. Draining large-volume PE led to a progressive increase in left ventricular end-diastolic volume index, left atrial volume index, right ventricular area, right atrial area, left ventricular ejection fraction, stroke volume, and tricuspid annular plane systolic excursion, both immediately (*P <* 0.05) and 24 h after drainage (*P <* 0.05). The cardiac diastolic measurement ratios of early-transmitral flow velocity to diastolic mitral annular velocity and myocardial performance index decreased significantly following drainage (*P <* 0.05). More parameters were influenced by left-sided PE drainage. The correlation between effusion volume and changes in echocardiographic measurements was not statistically significant.

**Conclusions:**

Improved preload, and systolic and diastolic function is pivotal for hemodynamic change after draining large PE volumes. Subjects experienced improved cardiac hemodynamics following PE drainage, underlining the beneficial therapeutic and subjective effects.

**Electronic supplementary material:**

The online version of this article (10.1186/s12890-018-0625-5) contains supplementary material, which is available to authorized users.

## Background

Large-volume pleural effusion (PE) is a common complication causing breathlessness, and patients often experience dramatic and immediate relief from dyspnea after therapeutic thoracentesis [1]. Although it is a well-recognized phenomenon, the detailed mechanism underlying witnessed improvements following drainage remains poorly understood [2]. The reasons for breathlessness caused by PE maybe multifactorial, but improvements in pulmonary mechanics do not explain the degree of relief following thoracentesis [3].

Previous studies have focused on the effects of PE on cardiovascular hemodynamics. Case reports have shown that PE can cause circulatory instability and hemodynamic compromise through a mechanism resembling cardiac tamponade [4, 5]. An early animal model study supported this phenomenon [6]. Other investigators have proposed that drainage after pleurocentesis may contribute to cardiac preload improvement and hemodynamic optimization in large-volume PE patients [7]. However, exactly when the hemodynamic change is most obvious remains unknown; therefore, evaluating the effect of drainage on the cardiovascular system at different points in time may be beneficial and may contribute to understanding the resulting dyspnea relief.

The aim of this prospective study was to assess the effects of drainage of large-volume PE and changes in cardiac function at different time points, then to compare the hemodynamic measurements between PE on different sides of the thorax.

## Methods

This study was approved by the Ethics Committee of Beijing Chaoyang Hospital and conducted in agreement with the Helsinki II declaration. Written informed consent was obtained from all participants. Approval was obtained from the Beijing Chao-Yang Hospital Institutional Review Board (approval number: 2016–2–19–9), and the clinical trial number is NCT02548221.

### Subjects

We assessed the eligibility of all PE patients who visit the department of Respiratory and Critical Care Medicine in Beijing Chaoyang Hospital and required pleural drainage for the treatment of breathlessness.

Inclusion criteria: 1. Subjects with symptomatic PE secondary to non-cardiac etiology who required pleural drainage as part of standard clinical management of the effusion. 2. No drainage was performed within 1 month before admission. 3. Written informed consent was obtained.

Exclusion criteria: 1) Subjects with physical weakness making drainage difficult to endure; 2) Subjects who had undergone drainage within 1 month before admission; 3) We excluded subjectswith pericardial effusion because the pericardial effusion may have confounded our results [8].

### Drainage procedure

All drainage procedures were performed by the same trained pulmonologist using standard protocol. Thoracentesis, indwelling pleural catheters,or medical thoracoscopy was chosen for pleural opening to perform large-volume drainage. Thoracentesis was performed with echocardiographic evaluation immediately after drainage, and a second echocardiographic examination was performed 24 h later. The medical thoracoscopy procedure and equipment have been described previously [9, 10]. Pleural biopsies were collected for diagnostic confirmation of the cause of the PE, and we recorded the PE characteristics, including the volume and appearance, etiology and details of any prior pleural procedures.

### Transthoracic echocardiography(TTE)

All subjects were evaluated by comprehensive TTEby Dr. Xiu-zhang LV and Qi-Zhe Cai according to the American Society of Echocardiography [11] at three time points: prior to drainage (baseline), immediately after, and 24 h after drainage. Both the two doctors were blinded to the three time points assignment. Echocardiographic images were acquired using an EPIQ 7C scanner (Philips Medical Systems, Bothell, WA) with a matrix-array transducer (X5–1). The specific methods are included in the Additional file 1.

Briefly, we used three-and two-dimensional quantification to quantify chamber volumes: end-diastolic and end-systolic left ventricular (LV)volume, ejection fraction and stroke volume (SV),left atrial, LV end-diastolic volume index (LVdVI), LV end-systolic volume index (LVsVI), and left atrial volume index (LAVI).These volume values were divided by the body surface index for each patient. Right ventricular (RV) and right atrial (RA) areas were evaluated in apical four-chamber views. Right ventricular systolic function was evaluated by fractional area change (FAC) and tricuspid annular plane systolic excursion (TAPSE). Mitral and tricuspid valve velocities were recorded as E, A, and TvE, TvA, respectively. E/A and TvE/A were the ratio of E over A and the ratio of TvE over TvA, respectively. Annular velocities of the mitral and tricuspid valves were analyzed by tissue Doppler image and recorded as Em, Am, and Et. E/Em and TvE/Et were the ratios of E over Em and TvE over Et, respectively. LV isovolumic relaxation time and RV myocardial performance index (MPI) were also recorded. Global left ventricular strain (GLS) and right ventricular free-wall strain (RVFWs) were derived from two-dimensional speckle tracking images.

### Statistical analysis

Comparisons were performed using Wilcoxon’s test according to the distribution of the data. We used Pearson’s χ^2^ test to test for differences in distribution of categorical variables between groups with PE on different sides of the thorax. Differences in parameters when comparing data immediately after thoracentesis or 24 h after thoracentesis with baseline were assessed using Wilcoxon’s test. To explore whether the parameter was maintained, enhanced, or lost 24 h after drainage, we compared data from immediately after drainage with the 24-h data using Wilcoxon’s test (paired difference test).To test for correlations among variables, we used Spearman’s correlation test, and we created a scatter plot to demonstrate the significant correlations. All *P*-values were two-sided, and *P* < 0.05 was considered statistically significant. All statistical analyses were performed using the Statistical Package for the Social Sciences (SPSS 13.0, Chicago, IL).

## Results

### Characteristics of the study population

Forty-seven subjects were included in the study from June 9, 2015 to September 18, 2016 in Beijing Chaoyang Hospital, and the side distribution of drained PE was 23 (48.9%)left-sided and 24 (51.1%)right-sided. There were no significant differences in baseline values for age, smoking status, medical history, body surface area, effusion volume, and effusion appearance between the two groups (Table 1). There were significant differences in gender and Nt-pro BNP between left- and right-sided effusion groups, with *P* < 0.022 and 0.004 respectively. Table 2 shows the etiologies of the PE.

**Table 1:** Subjects’ characteristics

Variables	Total	Lateral of effusion, n (%)		P* value
		Left	Right	
Subjects, n(%)	47 (100.0)	23 (48.9)	24 (51.1)	
Gender, n (%)				
Female	18 (38.3)	5 (21.7)	13 (54.2)	0.022
Male	29 (61.7)	18 (78.3)	11 (45.8)	
Age, yr., mean ± SD	56.4 ± 19.5	52.6 ± 19.0	63.1 ± 17.3	0.726
BSA, mean ± SD	1.73 ± 0.19	1.83 ± 0.16	1.63 ± 0.17	0.582
Smoking status, n (%)				
Current or previous smoker	18 (38.3)	6 (26.1)	12 (50.0)	0.092
Non-smoker	29 (61.7)	17 (73.9)	12 (50.0)	
Past history				
Tuberculosis	4 (8.5)	3 (13.0)	1 (4.2)	0.546
Malignancy	4 (8.5)	2 (8.7)	2 (8.3)	
NA	39 (83.0)	18 (78.3)	21 (87.5)	
Size of effusion, n (%)				
Small	8 (17.0)	3 (13.0)	5 (20.8)	0.696
Moderate	9 (19.1)	4 (17.4)	5 (20.8)	
Large	30 (63.9)	16 (69.6)	14 (58.3)	
volume of effusion, (ml)				
Total volume	2142.2 ± 1393.7	2194.6 ± 1511.1	2203.6 ± 1301.8	0.827
Immediate volume	1414.4 ± 727.9	1384.8 ± 982.0	1495.2 ± 947.6	0.744
24 h volume	943.6 ± 742.8	809.8 ± 787.2	708.4 ± 708.0	0.855
Effusion appearance, n (%)				
Bloody	20 (42.5)	11 (47.8)	9 (37.5)	0.474
Yellow	27 (57.5)	12 (52.2)	15 (62.5)	
Operation				
IPC	15 (31.9)	8 (34.8)	7 (29.2)	0.893
Thoracentesis	10 (21.3)	5 (21.7)	5 (20.8)	
thoracoscopic	22 (46.8)	10 (43.5)	12 (50.0)	
Pre Nt-pro-BNP (ng/ml)	221.3 ± 316.5	126.7 ± 176.6	320.2 ± 396.5	0.004

*Abbreviations*: *SD* standard deviation, *IPC* indwelling pleural catheter

P*, Wilcoxon’s test or Pearson’s χ^2^ test, *P* < 0.05

**Table 2:** Causes of pleural effusion

Cause		n, %
Malignancies		24 (51.1)
	Primary lung cancer	14 (29.8)
	Secondary carcinoma	2 (4.3)
	Mesothelioma	4 (8.5)
	Lymphoma	1 (2.1)
	Undetermined	3 (6.4)
Benign diseases		23 (48.9)
	Tuberculosis	17 (36.0)
	Non-specific pleurisy	2 (4.3)
	Other transudate	2 (4.3)
	Other exudates	2 (4.3)
Total		47 (100.0)

The mean volume of removed fluid was 2142.2 ± 1393.7 mL. We excluded 14subjectsbecause of a lack of data from: baseline (*n* = 6), immediately after drainage(*n* = 5), or 24 h after drainage(*n* = 3).Five subjects were also excluded because of atrial fibrillation (*n* = 1) or poor three-dimensional echocardiography data (*n* = 4). A final 28 subjects were enrolled with 13 left- and 15 right-sided PE. No Nt-pro BNP difference was found between left and right when looking at just 28 analyzed subjects (data not shown).

### Overall influence of PE on cardiac hemodynamics

The influence of PE on cardiac hemodynamics was evaluated by comparing differences in real-time parameters with echocardiographic measurements. The preload parameters, LVdVI, LAVI, RV area, and RA area increased significantly both immediately after drainage and 24 h after drainage (*P* < 0.05). LVsVI improved significantly only at 24 h after drainage. Similarly, the systolic measurements, LVEF, SV, and TAPSE, increased significantly at bothpost-drainage time points (*P* < 0.05). GLS and RVFWs increased 24 h after drainage (*P* < 0.05) but not immediately post-drainage (*P* > 0.05). In contrast, the LV diastolic measurements, E/Em and A (late transmitral flow velocity), decreased24 hours after drainage. MPI, representing RV diastolic function, decreased significantly(*P* < 0.05). Significant differences between values immediately after and 24-h after drainage were seen for LVdVI, SV, and TAPSE(*P* < 0.05). The other variables showed no significant changes during the measurement periods (see Table 3).

**Table 3:** Real-time echocardiographic changes in hemodynamics before and after pleural drainage

Parameters	Effusion (*n* = 28)					
	Before	Immediately After	*P* ^a^	24 h After	*P* ^b^	*P* ^c^
Left Heart						
LVdVI, (ml/m^2^)	31.9 (24.3–42.8)	35.3 (31.3–44.9)	< 0.001	36.7 (31.1–48.2)	< 0.001	0.019
LVsVI, (ml/m^2^)	11.3 (9.4–16.7)	12.2 (10.2–16.3)	0.220	12.2 (10.3–15.2)	< 0.001	0.264
LAVI, (ml/m^2^)	22.8 (17.2–26.0)	26.5 (19.1–29.7)	0.018	27.4 (22.9–32.0)	< 0.001	0.234
Right Heart						
RV area, (cm^2^)	14.1 (12.5–18.9)	17.3 (15.2–18.9)	0.036	17.3 (15.0–18.5)	0.011	0.782
RA area, (cm^2^)	11.8 (9.9–14.9)	14.1 (11.3–16.3)	0.016	14.2 (11.3–15.6)	0.018	0.727
Left ventricle						
LVEF, (%)	62.0 (59.3–66.8)	65.0 (63.0–68.8)	0.030	68.5 (64.0–71.0)	0.008	0.071
SV, (ml)	36.0 (27.3–47.5)	41.0 (35.5–49.8)	< 0.001	42.5 (38.0–52.6)	< 0.001	0.006
Sm, (cm/s)	8.2 (7.5–9.7)	8.0 (7.5–9.3)	0.952	9.0 (8.0–9.6)	0.137	0.055
GLS, (%)	19.0 (18.0–20.0)	19.5 (18.0–21.0)	0.086	21.0 (18.0–22.9)	< 0.001	0.029
Right ventricle						
FAC, (%)	45.5 (38.3–52.0)	48.0 (45.0–57.0)	0.065	50.0 (40.0–55.0)	0.347	< 0.001
TAPSE, (cm)	18.0 (17.0–19.8)	20.1 (19.0–22.0)	< 0.001	22.4 (19.5–24.8)	0.001	< 0.001
RVFWs, (%)	18.0 (17.6–19.3)	19.1 (18.3–21.5)	0.137	19.3 (18.3–21.1)	0.018	0.603
Left ventricle						
IVRT, (s)	106.0 (106.0–127.0)	113.0 (106.0–123.0)	0.601	118.0 (106.0–135.3)	0.310	0.649
E, (cm/s)	74.5 (67.8–85.0)	70.7 (64.3–76.8)	0.285	64.8 (56.8–82.4)	0.178	0.683
A, (cm/s)	85.5 (63.8–113.8)	91.9 (58.7–103.3)	0.493	80.8 (48.9–93.4)	0.019	0.058
E/A	0.9 (0.7–1.1)	0.8 (0.7–1.3)	0.546	0.9 (0.7–1.3)	0.260	0.157
Em, (cm/s)	7.0 (6.1–9.8)	7.4 (6.3–8.7)	0.819	8.4 (6.2–9.3)	0.170	0.170
Am, (cm/s)	10.4 (8.9–11.7)	10.9 (10.0–12.1)	0.390	11.0 (10.4–12.6)	0.334	0.309
E/Em	10.9 (7.1–13.6)	9.8 (7.3–11.0)	0.419	9.1 (6.4–10.6)	0.023	0.095
Right ventricle						
MPI	0.7 (0.6–0.7)	0.5 (0.5–0.7)	< 0.001	0.5 (0.4–0.6)	0.002	0.585
TvE, (cm/s)	54.0 (45.0–65.3)	57.0 (50.3–65.9)	0.536	53.0 (46.2–64.8)	0.543	0.065
TvA, (cm/s)	51.5 (40.8–60.3)	48.7 (40.8–60.3)	1.000	41.8 (35.9–51.5)	0.069	0.027
TvE/A	1.1 (0.8–1.3)	1.2 (0.8–1.4)	0.619	1.3 (1.1–1.4)	0.122	0.265
TvE/Et	4.2 (3.6–5.8)	4.5 (3.1–4.9)	0.339	3.5 (2.8–5.4)	0.264	0.406

*Abbreviations*: *LVdVI* Left ventricular end diastolic volume index, *LVsVI* left ventricular end-systolic volume index, *LAVI* left atrial volume index, *RV* area, right ventricular area, *RA* area, right atrial area, *LVEF* left ventricular ejection fraction, *SV* stroke volume, *Sm* systolic mitral annular velocity, *GLS* global left ventricular strain, *FAC* fractional area change, *TAPSE*, tricuspid annular plane systolic excursion, *RVFWs* right ventricular free-wall strain, *E* early-transmitralflow velocity, *A* late-transmitral flow velocity, *E/A*, ratio of early- to late-transmitral flow velocity, Em, early-diastolic mitral annular velocity, *Am* late-diastolic mitral annular velocity, *E/Em* ratio of early-transmitral flow velocity to diastolic mitral annular velocity, *MPI*, myocardial performance index, *TvE* early-transtricuspid flow velocity, *TvA* late-transtricuspid flow velocity;TvE/A, ratio of early- to late-transtricuspid flow velocity, *TvE/Et*, ratio of early-transtricuspid flow velocity to diastolic tricuspid annular velocity

*P^a^ < 0.05 compared with the corresponding parameters before and immediately after drainage; *P*^b^ < 0.05 compared with the corresponding parameters before and 24 h after drainage;*P*^c^ < 0.05 compared with the corresponding value immediately after drainage and the value 24 h after drainage

### Subgroup analysis of PE influence on cardiac hemodynamics based on left or right side

To explore whether different PE on different sides of the thorax has a distinct influence on cardiac function, we divided subjects into the PE subgroups, left-sided (*n* = 13) and right-sided (*n* = 15). Comparisons of the echocardiographic measurements in the subgroups appeared in Additional file 2 Tables S1, S2 and S3. Significant changes were observed for LVdVI, LVsVI, LAVI, RV area, SV, TAPSE, and MPI when data for both groups were pooled. Increases in LVEF, FAC, RVFWs, and TvE/A, and decreases in E/Em and TvA were significant in the left-sided group(*P* < 0.05). We also saw significant improvements in RA area and GLS after drainage in the right-sided group (*P* < 0.05).

**Fig. 1:**
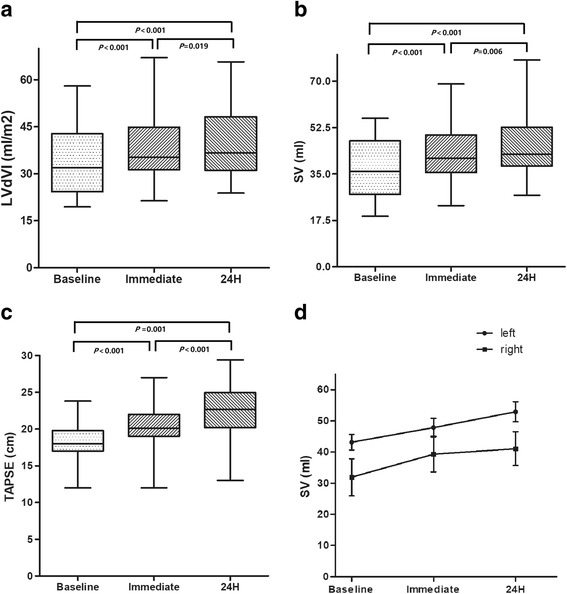
**a**–**c** shows the changes in LVdVI, stroke volume, and TAPSE at baseline, immediately after, and 24 h after drainage for the entire study population. **d** shows the changes instroke volume in the left- and right-sided effusion subgroups. LVdVI, left ventricular end diastolic volume index; SV, stroke volume; TAPSE, tricuspid annular plane systolic excursion

For left-sided PE, LVdVI, SV, TAPSE, RVFWs, MPI, and TvE/A changed significantly both immediately after and 24 h after drainage. For example, for SV (Fig. 1d), the median value increased from 42.0 ml at baseline to 46.0 ml immediately after drainage (*P*^*a*^ = 0.002), then to 51.4 ml 24 h after drainage (*P*^*b*^ = 0.002, *P*^*c*^ = 0.007).However, the median and quartile FAC ranges increased from 62.0% (60.0%–66.0%)of baseline to 64.0% (63.0%–65.0%) of baseline only immediately after drainage (*P*^*a*^ = 0.043); no changes were seen 24 h after drainage (*P*^*b*^ = 0.308, *P*^*c*^ = 0.107). Other measurements in the left-sided group also changed very little 24 h after drainage including LVsVI, LAVI, RV area, LVEF, E/Em, and TvA.

In the right-sided group, some measurements, including LVsVI, LAVI, and GLS increased only 24 h after drainage and were unchanged immediately after drainage. MPI changed very little immediately after drainage. Also in the right-sided group, the median RV area increased from 13.2cm^2^ at baseline to 18.2cm^2^ immediately after drainage (*P*^*a*^ = 0.005) then decreased to 17.3cm^2^24 h after drainage (*P*^*b*^ = 0.008); no significant difference was seen between the immediately-after and 24 h-after data (*P*^*c*^ = 0.156).

### Correlation between PE drainage volume and changes in echocardiographic measurements

Additional file 2 Table S4 shows the correlation between PE drainage volume and changes in echocardiographic measurements. We found no consistent relationship between PE volume and changes in echocardiographic measurements between different sides. We did see a positive influence of effusion volume on RA area parameters 24 h after drainage(*r* = 0.501)but without statistical significance (*P* = 0.057).

## Discussion

Our research was a comprehensive study to discuss the relationship between PE drainage and echo cardiovascular parameters, including preload parameters, and systolic and diastolic measurements.

This study showed that drainage of large PE volumes led to an immediate increase in LVdVI, LAVI, RV area, and RA area for all enrolled subjects. While previous studies showed that removing large PE volumes may cause relative hypovolemia secondary to fluid redistribution to the pleural cavity [12], our results did not support these findings. More studies have shown improved left cardiac preload in both human [7] and porcine models [6], similar to our results. This improvement may be associated with the underlying mechanism in which PE increases pulmonary arterial resistance and pulmonary wedge pressure through vessel compression; RV afterload then increases and LV preload decreases. PE drainage may attenuate RV afterload and amplify LV preload. With lung expansion and recovery of collapsed alveoli, RV afterload decreases further while LV preload subsequently increases, which was confirmed by our results. Preload parameters, including LVdVI,increased24 h after drainage compared with immediately after drainage. This change may imply that improved hemodynamics may occur immediately and could be amplified with lung expansion, and may explain why the increased LVsVI reached statistical significance only 24 h after drainage.

Improved RA and RV areaimply that PE maybe responsible for collapse of the right heart, which was seen in a porcine model measuring central venous pressure(CVP), a surrogate marker for RV transmural pressure [6]. PE decreased CVP and right-lateral transmural pressure, which meant a decrease in RV end-diastolic volume. Therefore, PE drainage may lead to increased RV preload, also shown in several previous studies. Sadaniantz and co-authors showed that PE led to RA collapse [8], and Vaska and co-authors suggested that large-volume PE can increase intrapericardial pressure and cause RV diastolic collapse [13]. Therefore, based on Frank-Starling’slaw, if RVFAC increases, then heart beats after this effect can transmit to the LV. Although changes in FAC did not reach statistical significance, the median value tended to increase, in our study.

Also in our study, systolic function measured by LVEF, SV, and TAPSE improved after drainage, similar to end-diastolic volume(preload). This phenomenon can be explained by the Frank-Starling mechanism in that increased LV preload can result in increased SV within a range. However, ina study by Hermansen and co-authors, ejection fraction did not change after pleurocentesis [7]. Two underling reasons may explain this difference. First, the study suggested that changes in LV end-diastolic volume and LV end-systolic volume were inconstant. Although both LVdVI and LVsVI increased following drainage, LVdVI increase may have been greater than that of LVsVI, which causes a greater SV(SV = LVdV − LVsV), tending to increase LVEF (LVEF=SV/LVdV). Second, increased GLS and RVFWs may reflect increased myocardial contractility to increase both LVEF and FAC.

We interpreted decreased A, E/Em, and MPI as improved diastolic function. A previous study demonstrated that A, E, TvA, and TvE of tricuspid and mitral valves all decreased after thoracocentesis with unreliable results [14]. However, we observed a significant change for A only 24 h after drainage. This difference maybe because of poor echo windows, which reduced the accuracy of measurements in the previous study. A and E/Em are two of the principal deflections for assessing transmitral velocity patterns. Therefore, decreasing values can be used to predict decreasing LV filling pressures, which represent improved LV diastolic function. Heart rate may have an impact on transmitral flow pattern; therefore, we added MPI, in our study. Decreased MPI means improved RV diastolic function, and this fact supports the argument that PE may generate diastolic dysfunction.

In the study, the statistically-significant changes of some echo parameters were fairly small, such as LVEF, TAPSE. It may be asymptomatic and may do not have clinically meaning in healthy subjects who do not suffer from large volume of PE. But for patients with large volume of PE, the change may have some sub-clinical significance. This findings regarding the large-volume drainage –induced changes in echocardiographic parameters need to be confirmed in much larger and more heterogenous collectives of patients.

When dividing the population into subgroups of left and right sides of the thorax, we saw interesting results. Most of the preload and systolic parameters, except RA area and LVEF, improved for both sides. More parameters were influenced by left lateral PE, possibly because the presence of large-volume PE may have dissimilar impact on compliance on the different ventricles secondary to the different anatomical structures in the left and right heart. A recent study in ventilated animals investigated the relationship between cardiac impedance and intrathoracic pressure. PE decreased lung compliance and simultaneously increased chest wall compliance [15], but the authors did not discuss hemodynamic variables and ventricular compliance. Regarding the hemodynamic effects of PE, several studies demonstrated that large-volume PE may decrease compliance in the left ventricle while increasing pulmonary capillary wedge pressure and CVP, and reducing cardiac outputand arterial pressure [16–18]. Brochand co-authors suggested that bilateral PE does not affect the ability of certain dynamic variables to predict fluid responsiveness in a porcine model [6]. However, neither of these studies compared the differences of echocardiographic measurements between two sides of PE.

We saw no dose-response relationship between PE volume and changes in cardiovascular hemodynamic measurements, which we did not expect, and which may reflect several important underlying reasons. First, the effect of PE on cardiovascular measurements may have many confounding factors, which can be controlled only in animal models through invasive measures. Second, the severity of breathlessness was poorly correlated with effusion volume. Previous studies concluded that effusion volume does not influence gas exchange and changes inPaO_2_ [19, 20], and a similar relationship between effusion volume and hemodynamic changes may also exit. Therefore, further studies with larger sample sizes are needed to determine whether PE volume definitively affects hemodynamics.

Due to poor quality data, some enrolled PE subjects were excluded from the final analysis. So we made a comparison between the enrolled and excluded subjects in baseline characteristics (data not shown) and found no significant differences between the groups. There is no apparent choosing bias in the methodology and the excluded subjects does not affect the final analysis.

## Conclusions

Our study maybe one of the most comprehensive studies to address changes in basic hemodynamic determinants induced by PE drainage in people, based on TTE. Our results implied significant improvement in cardiac preload and systolic function with substantial improvement in diastolic function following drainage. To our knowledge, ours is the first study to divide PE subjects into left- and right-sided subgroups and to show that PE on different sides may have distinct influences on cardiac hemodynamics. Our findings do not support the hypothesis that higher drainage volumes cause a linear improvement in cardiac function. Our results indicated that improved cardiac hemodynamics are a considerable contributor to the underlying mechanisms causing dyspnea in PE patients. These findings may help illustrate how PE influences cardiac hemodynamics, which then helps determine the therapeutic approach to PE.

## Additional files

Additional file 1: Transthoracic echo cardiography(TTE). The specific methods describing how the cardiocalic paremeters were measured or calculated using the transthoracic echocardiography. (DOCX 14 kb)

Additional file 2: **Table S4.** The changes of pre-load parameters by echocardiographic findings before and after drainage of subgroups of pleural effusions. Significant changes were observed on LVdVI for both laterals at both immediate and 24 hours drainage. But RV area and RA area were only observed to increase in the right group without alterations in the left PE. No significant differences were seen between the immediately after drainage and 24 hours after drainage data (with *P*^c^ > 0.05). **Table S5.** The changes of systolic functions by echocardiographic findings before and after drainage of subgroups of pleural effusions. Some systolic measurements, SV and TAPSA changed on both laterals at both time points. While increases in LVEF, FAC and RVFWs were significant only in the left group. In the right group, GLS increased only at 24 hours after drainage. **Table S6** The changes of diastolic functions by echocardiographic findings before and after drainage of subgroups of pleural effusions. The diastolic parameter, MPI changed on both sides of PE. The increase in TvE/A and decreases in E/Em and TvA were significant merely in the left group. (DOCX 35 kb)

## Abbreviations

A: Late-peak mitral valve velocity
Am: Late-diastolic mitral annular velocity
E: Early-transmitral flow velocity
E/A: Ratio of early to late transmitral flow velocity
E/Em: Ratio of early transmitral flow velocity to diastolic-mitral annular velocity
EF: Ejection fraction
Em: Early-diastolic mitral annular velocity
FAC: Fractional area change
GLS: Global left ventricular strain
IVRT: Isovolumic relaxation time
LA: Left atrial
LAVI: Left atrial volume index
LV: Left ventricular
LVdVI: Left ventricular end-diastolic volume index
LVEF: Left ventricular ejection fraction
LVsVI: Left ventricular end-systolic volume index
MPI: Myocardial performance index
PE: Pleural effusion
RA area: Right atrial area
RV area: Right ventricular area
RVFWs: Right ventricular free wall strain
SV: Stroke volume
TAPSE: Tricuspid annular plane systolic excursion
TTE: Transthoracic echocardiography
TvA: Late-transtricuspid flow velocity
TvE: Early-transtricuspid flow velocity
TvE/A: Ratio of early to late transtricuspid flow velocity
TvE/Et: Ratio of early-transtricuspid flow velocity to diastolic-tricuspid annular velocity

## References

[1] Mitrouska I, Klimathianaki M, Siafakas NM (2004). Effects of pleural effusion on respiratory function. Can Respir J.

[2] Estenne M, Yernault JC, De Troyer A (1983). Mechanism of relief of dyspnea after thoracocentesis in patients with large pleural effusions. Am J Med.

[3] Light RW, Stansbury DW, Brown SE (1986). The relationship between pleural pressures and changes in pulmonary function after therapeutic thoracentesis. Am Rev Respir Dis.

[4] Kaplan LM, Epstein SK, Schwartz SL, Cao QL, Pandian NG (1995). Clinical, echocardiographic, and hemodynamic evidence of cardiac tamponade caused by large pleural effusions. Am J Respir Crit Care Med.

[5] Kopterides P, Lignos M, Papanikolaou S, Papadomichelakis E, Mentzelopoulos S, Armaganidis A (2006). Pleural effusion causing cardiac tamponade: report of two cases and review of the literature. Heart Lung.

[6] Broch O, Gruenewald M, Renner J, Meybohm P, Schöttler J, Heß K (2013). Dynamic and volumetric variables reliably predict fluid responsiveness in a porcine model with pleural effusion. PLoS One.

[7] Hermansen JF, Juhl-Olsen P, Frederiksen CA, Christiansen LK, Hørlyck A, Sloth E (2014). Drainage of large pleural effusions increases left ventricular preload. J Cardiothorac Vasc Anesth.

[8] Sadaniantz A, Anastacio R, Verma V, Aprahamian N (2003). The incidence of diastolic right atrial collapse in patients with pleural effusion in the absence of pericardial effusion. Echocardiography.

[9] Wang F, Wang Z, Tong Z, Xu L, Wang X, Wu Y (2015). A pilot study of autofluorescence in the diagnosis of pleural disease. Chest.

[10] Wang XJ, Yang Y, Wang Z, Xu LL, Wu YB, Zhang J (2015). Efficacy and safety of diagnostic thoracoscopy in undiagnosed pleural effusions. Respiration.

[11] Lang RM, Badano LP, Mor-Avi V, Afilalo J, Armstrong A, Ernande L (2015). Recommendations for cardiac chamber quantification by echocardiography in adults: an update from the American Society of Echocardiography and the European Association of Cardiovascular Imaging. J Am Soc Echocardiogr.

[12] Light RW, Jenkinson SG, Minh VD, George RB (1980). Observations on pleural fluid pressures as fluid is withdrawn during thoracentesis. Am Rev Respir Dis.

[13] Vaska K, Wann LS, Sagar K, Klopfenstein HS (1992). Pleural effusion as a cause of right ventricular diastolic collapse. Circulation.

[14] Chidambaram S, Sangareddi V, Ganesan G, Dhandapani VE, Ravi MS, Meenakshi K (2013). An echocardiographic assessment of cardiovascular hemodynamics in patients with large pleural effusion. Indian Heart J.

[15] Graf J, Formenti P, Santos A, Gard K, Adams A, Tashjian J (2011). Pleural effusion complicates monitoring of respiratory mechanics. Crit Care Med.

[16] Ahmed SH, Ouzounian SP, Dirusso S, Sullivan T, Savino J, Del Guercio L (2004). Hemodynamic and pulmonary changes after drainage of significant pleural effusions in critically ill, mechanically ventilated surgical patients. J Trauma.

[17] Lan CC, Hsu HH, Wu CP, Lee SC, Peng CK, Chang H (2014). Influences of pleural effusion on respiratory mechanics, gas exchange, hemodynamics, and recruitment effects in acute respiratory distress syndrome. J Surg Res.

[18] Nishida O, Arellano R, Cheng DC, DeMajo W, Kavanagh BP (1999). Gas exchange and hemodynamics in experimental pleural effusion. Crit Care Med.

[19] Chang SC, Shiao GM, Perng RP (1989). Postural effect on gas exchange in patients with unilateral pleural effusions. Chest.

[20] Romero S, Martin C, Hernandez L, Arriero JM, Benito N, Gil J (1995). Effect of body position on gas exchange in patients with unilateral pleural effusion: influence of effusion volume. Respir Med.

