# Supplementary material for: Improved heart hemodynamics after draining large-volume pleural effusion: a prospective cohort study
Source: BMC Pulm Med. 2018 Apr 25;18:62. doi: 10.1186/s12890-018-0625-5 (PMC5921556; doi:10.1186/s12890-018-0625-5)
Supplement: Supplementary file 1 — Transthoracic echo cardiography(TTE). The specific methods describing how the cardiocalic paremeters were measured or calculated using the transthoracic echocardiography. (DOCX 14 kb) [file 12890_2018_625_MOESM1_ESM.docx]

**Transthoracic echocardiography(TTE)**

To assess the accurate left ventricular (LV) volume, we collected full-volume 3D data sets. 3D manual measurement was performed using software of QLAB-3DQ (Philips Healthcare). End-diastolic and end-systolicLVvolume, EF and SVwere derived from the 3D Quantification Analysis. Left atrial volume was calculated using the Biplane method of disks.RV and RA areas were traced from apical four-chamber view at end-diastole and end-systole respectively. Fractional area change (FAC) were calculated by formula FAC= (end-diastolic RV area—end-systolic RV area)*100%/ end-diastolic RV area. Tricuspid annular plane systolic excursion (TAPSE) was measured under M-mode by placing sample line through lateral tricuspid annulus.Velocity of mitral valve and tricuspid valve were recorded as early peak mitral valve velocity (E), late peak mitral valve velocity (A), early peak tricuspid valve velocity (TvE) and late peak tricuspid valve velocity (TvA). E/A and TvE/A were the ratio of E over A and ratio of TvE over TvA.Tissue Doppler image analysis of the annular velocity of mitral valve and tricuspid valve were obtained by placing sample line across the septal and lateral annulus. Em was the average of septal and lateral mitral annular early diastolic peak velocity. E/Em was the ratio of early diastolic peak mitral valve velocity over early diastolic peak mitral annular velocity. Am wasthe average of septal and lateral mitral annular late diastolic peak velocity. Et was the early diastolic lateral tricuspid annular velocity. TvE/Et was the ratio of early tricuspid velocity overlateral tricuspid annular early diastolic peak velocity.Left ventricular isovolumic relaxation time (IVRT)was measured as the time duration from the end of systolic left ventricular outflow wave to the beginning of early diastolic mitral wave. Myocardial performance index (MPI) was calculated according the formula (right ventricular isovolumic relaxation time + isovolumic contraction time)/ejection time. All these three time durations were derived from wave pattern of Et. Global left ventricular strain (GLS) was the average strain of each left ventricular segmenton apical four-chamber, two-chamber and three-chamber view.Right ventricular free wall strain (RVFWs) was the average strain of right ventricular free wall segments. The difference of each parameter was calculated as the value immediate after or 24 hours after drainage minus the value before drainage.
