# Supplementary material for: Improved heart hemodynamics after draining large-volume pleural effusion: a prospective cohort study
Source: BMC Pulm Med. 2018 Apr 25;18:62. doi: 10.1186/s12890-018-0625-5 (PMC5921556; doi:10.1186/s12890-018-0625-5)
Supplement: Supplementary file 2 — Table S4. The changes of pre-load parameters by echocardiographic findings before and after drainage of subgroups of pleural effusions. Significant changes were observed on LVdVI for both laterals at both immediate and 24 hours drainage. But RV area and RA area were only observed to increase in the right group without alterations in the left PE. No significant differences were seen between the immediately after drainage and 24 hours after drainage data (with Pc > 0.05). Table S5. The changes of systolic functions by echocardiographic findings before and after drainage of subgroups of pleural effusions. Some systolic measurements, SV and TAPSA changed on both laterals at both time points. While increases in LVEF, FAC and RVFWs were significant only in the left group. In the right group, GLS increased only at 24 hours after drainage. Table S6 The changes of diastolic functions by echocardiographic findings before and after drainage of subgroups of pleural effusions. The diastolic parameter, MPI changed on both sides of PE. The increase in TvE/A and decreases in E/Em and TvA were significant merely in the left group. (DOCX 35 kb) [file 12890_2018_625_MOESM2_ESM.docx]

**Table S4.The changes of pre-load parameters by echocardiographic findings before and after drainage of subgroups of pleural effusions.**

| Parameters | Left Effusion (n = 13) | | | | | |  | Right Effusion (n = 15) | | | | |  |
| --- | --- | --- | --- | --- | --- | --- | --- | --- | --- | --- | --- | --- | --- |
|  | Before | Immediately After | *P*^a^ | 24 hours After | *P*^b^ | *P*^c^ |  | Before | Immediately After | *P*^a^ | 24 hours After | *P*^b^ | *P*^c^ |
| Left Heart |  |  |  |  |  |  |  |  |  |  |  |  |  |
| LVdVI, (ml/m^2^) | 39.2  (33.2-47.1) | 42.4  (34.0-50.4) | 0.036 | 44.4  (36.7-54.0) | 0.013 | 0.084 |  | 26.0  (22.0-32.3) | 32.4  (25.5-36.7) | 0.001 | 32.4  (30.4-37.8) | 0.001 | 0.087 |
| LVsVI, (ml/m^2^) | 14.9  (11.8-18.9) | 16.0  (12.9-18.0) | 0.463 | 14.4  (11.8-15.8) | 0.028 | 0.028 |  | 9.6  (8.0-10.7) | 10.3  (7.7-12.0) | 0.331 | 11.7  (8.5-14.1) | 0.001 | 0.683 |
| LAVI, (ml/m^2^) | 23.3 (16.0-25.6) | 26.1  (18.6-29.4) | 0.152 | 25.6  (22.4-31.4) | 0.002 | 0.388 |  | 22.6  (17.7-26.2) | 27.7  (20.8-38.2) | 0.053 | 28.4  (23.9-34.6) | 0.001 | 0.320 |
| Right Heart |  |  |  |  |  |  |  |  |  |  |  |  |  |
| RV area, (cm^2^) | 14.5  (13.8-18.0) | 15.6  (12.0-18.1) | 0.889 | 17.8  (13.8-19.1) | 0.013 | 0.158 |  | 13.2  (12.2-18.9) | 18.2  (15.3-20.2) | 0.005 | 17.3  (15.0-17.8) | 0.008 | 0.156 |
| RA area, (cm^2^) | 12.4  (11.0-13.8) | 13.9  (10.9-15.9) | 0.346 | 13.7  (11.3-15.5) | 0.224 | 0.861 |  | 10.6  (9.3-15.0) | 14.8  (12.1-16.6) | 0.031 | 14.4  (11.3-15.6) | 0.024 | 0.572 |

Abbreviation: LVdVI, left ventricular end diastolic volume index; LVsVI, left ventricular end systolic volume index; LAVI, left atrial volume index;RV area, right ventricular area; RA area, right atrial area; LVEF, left ventricular ejection fraction; SV, stroke volume; Sm, systolic mitral annular velocity; GLS, global left ventricular strain; FAC, fractional area change; TAPSE, tricuspid annular plane systolic excursion; RVFWs, right ventricular free wall strain. E, early transmitral flow velocity; A, late transmitral flow velocity; E/A, ratio of early to late transmitral flow velocity; Em, early diastolic mitral annular velocity; Am, late diastolic mitral annular velocity; E/Em, ratio of early transmitral flow velocity to diastolic mitral annular velocity; MPI, myocardial performance index; TvE, early transtricuspid flow velocity; TvA, late transtricuspid flow velocity;TvE/A, ratio of early to late transtricuspid flow velocity; TvE/Et, ratio of early transtricuspid flow velocity to diastolic tricuspid annular velocity.

**P*^a^< 0.05 compared with the corresponding parameters before and immediately after drainage. *P*^b^ < 0.05 compared with the corresponding parameters before and 24 hours after drainage. *P*^c^< 0.05 compared with the corresponding value immediately after drainageand the value 24 hours after drainage.

| **Table S5:The changes of systolic functions by echocardiographic findings before and after drainage of subgroups of pleural effusions.** | | | | | | | | | | | | | |
| --- | --- | --- | --- | --- | --- | --- | --- | --- | --- | --- | --- | --- | --- |
| Parameters |  | Left Effusion (n = 13) | | | | |  | Right Effusion (n = 15) | | | | |  |
|  | Before | Immediately After | *P*^a^ | 24 hours After | *P*^b^ | *P*^c^ |  | Before | Immediately After | *P*^a^ | 24 hours After | *P*^b^ | *P*^c^ |
| Left ventricle |  |  |  |  |  |  |  |  |  |  |  |  |  |
| LVEF, (%) | 62.0  (59.5—65.0) | 64.0  (61.5-65.5) | 0.194 | 69.0  (65.5-71.0) | 0.003 | 0.010 |  | 63.0  (59.0-71.0) | 68.0  (64.0-70.0) | 0.083 | 67.0  (63.0-71.0) | 0.280 | 0.925 |
| SV, (ml) | 42.0  (36.0-53.0) | 46.0  (38.5-56.5) | 0.002 | 51.4  (43.0-61.0) | 0.002 | 0.007 |  | 28.0  (25.0-44.0) | 38.0  (32.0-46.0) | 0.001 | 38.1  (36.0-42.9) | 0.001 | 0.278 |
| Sm, (cm/s) | 9.0  (7.9-11.0) | 8.0  (7.8-9.6) | 0.146 | 9.0  (7.8-11.5) | 0.443 | 0.046 |  | 7.8  (7.2-9.0) | 8.0  (7.4-9.3) | 0.139 | 9.0  (8.0-9.5) | 0.140 | 0.572 |
| GLS, (%) | 19.0  (17.0-21.0) | 19.0  (17.5-21.0) | 0.723 | 21.0  (18.0-22.8) | 0.073 | 0.033 |  | 19.0  (18.0-20.0) | 20.0  (18.0-21.0) | 0.069 | 21.0  (18.0-23.0) | 0.003 | 0.263 |
| Right ventricle |  |  |  |  |  |  |  |  |  |  |  |  |  |
| FAC, (%) | 44.0  (35.5-52.0) | 54.0  (46.0-58.5) | 0.043 | 50.5  (34.5-57.5) | 0.308 | 0.107 |  | 47.0  (42.0-54.0) | 47.0  (43.8-55.3) | 0.638 | 49.0  (45.0-53.0) | 0.660 | 0.706 |
| TAPSE, (cm) | 19.0  (18.0-19.8) | 22.0  (20.1-23.0) | 0.005 | 23.3  (22.0-25.0) | 0.007 | 0.367 |  | 17.3  (16.5-18.9) | 19.0  (17.5-21.5) | 0.033 | 21.0  (18.3-23.0) | 0.041 | 0.116 |
| RVFWs, (%) | 18.0  (17.518.2) | 20.0  (19.0-21.4) | 0.036 | 19.3  (18.3-20.0) | 0.005 | 0.541 |  | 18.5  (17.9-19.8) | 19.0  (17.5-21.5) | 0.975 | 19.5  (18.5-21.1) | 0.638 | 0.239 |

Abbreviation: LVEF, left ventricular ejection fraction; SV, stroke volume; Sm, systolic mitral annular velocity; GLS, global left ventricular strain; FAC, fractional area change; TAPSE, tricuspid annular plane systolic excursion; RVFWs, right ventricular free wall strain.

**P*^a^< 0.05 compared with the corresponding parameters before and immediately after drainage. *P*^b^ < 0.05 compared with the corresponding parameters before and 24 hours after drainage. *P*^c^< 0.05 compared with the corresponding value immediately after drainageand the value 24 hours after drainage.

**Table S6.The changes of diastolic functions by echocardiographic findings before and after drainage of subgroups of pleural effusions.**

| Parameters |  | Left Effusion (n = 13) | | | | |  |  | Right Effusion (n = 15) | | | |  |
| --- | --- | --- | --- | --- | --- | --- | --- | --- | --- | --- | --- | --- | --- |
|  | Before | Immediately After | *P*^a^ | 24 hours After | *P*^b^ | *P*^c^ |  | Before | Immediately After | *P*^a^ | 24 hours After | *P*^b^ | *P*^c^ |
| Left ventricle |  |  |  |  |  |  |  |  |  |  |  |  |  |
| IVRT, (s) | 111.5  (106.0-126.0) | 113.0  (106.0-123.0) | 0.527 | 107.5  (106.0-124.3) | 0.610 | 0.779 |  | 109.5  (95.0-124.8) | 109.5  (100.2-157.8) | 0.374 | 120.0  (109.0-130.0) | 0.379 | 0.432 |
| E, (cm/s) | 75.0  (72.0-85.0) | 75.7  (66.0-84.0) | 0.552 | 68.0  (58.7-80.0) | 0.055 | 0.311 |  | 70.0  (66.0-85.0) | 68.0  (61.1-74.4) | 0.346 | 74.0  (57.3-85.3) | 0.975 | 0.594 |
| A, (cm/s) | 77.0  (63.0-96.0) | 68.9  (56.5-95.0) | 0.363 | 56.3  (43.4-91.0) | 0.055 | 0.108 |  | 82.0  (57.0-100.0) | 82.0  (70.3-101.0) | 0.972 | 82.8  (58.4-95.0) | 0.184 | 0.315 |
| E/A | 1.0  (0.7-1.3) | 1.0  (0.8-1.2) | 0.753 | 1.0  (0.9-1.4) | 0.208 | 0.327 |  | 0.9  (0.6-1.1) | 0.7  (0.7-1.3) | 0.712 | 0.8  (0.7-1.4) | 0.776 | 0.334 |
| Em, (cm/s) | 7.0  (6.4-10.5) | 7.4  (6.3-10.0) | 0.894 | 8.1  (7.0-10.5) | 0.213 | 0.327 |  | 8.6  (6.2-10.0) | 7.6  (6.8-10.1) | 0.776 | 8.9  (7.0-10.4) | 0.443 | 0.286 |
| Am, (cm/s) | 9.6  (8.9-11.5) | 10.0  (8.1-10.6) | 0.555 | 10.4  (9.0-11.1) | 0.656 | 0.074 |  | 10.4  (8.8-11.8) | 10.8  (9.3-14.0) | 0.065 | 10.7  (9.8-11.6) | 0.378 | 0.755 |
| E/Em | 9.3  (7.1-12.3) | 9.7  (6.7-11.4) | 0.506 | 8.3  (6.2-9.9) | 0.036 | 0.036 |  | 10.2  (7.0-11.5) | 8.2  (6.6-10.5) | 0.427 | 7.7  (6.8-9.9) | 0.258 | 0.778 |
| Right ventricle |  |  |  |  |  |  |  |  |  |  |  |  |  |
| MPI | 0.7  (0.6-0.8) | 0.6  (0.4-0.7) | 0.009 | 0.5  (0.5-0.6) | 0.011 | 0.552 |  | 0.6  (0.5-0.6) | 0.5  (0.4—0.6) | 0.009 | 0.5  (0.4-0.5) | 0.078 | 0.650 |
| TvE, (cm/s) | 51.0  (43.0-64.0) | 58.0  (51.0-64.0) | 0.230 | 55.8  (50.9-60.2) | 0.386 | 0.552 |  | 58.0  (51.0-64.0) | 58.0  (48.3-69.0) | 0.925 | 57.0  (46.5-64.0) | 0.173 | 0.088 |
| TvA, (cm/s) | 54.0  (49.5-64.5) | 48.0  (40.0-53.0) | 0.110 | 44.1  (38.8-52.0) | 0.013 | 0.345 |  | 44.5  (39.0-52.0) | 51.0  (46.3-60.8) | 0.167 | 46.1  (36.9-57.2) | 0.925 | 0.031 |
| TvE/A | 0.9  (0.8-1.1) | 1.3  (1.1-1.4) | 0.013 | 1.3  (1.2-1.3) | 0.006 | 0.701 |  | 1.2  (1.1-1.5) | 1.1  (0.8-1.4) | 0.140 | 1.2  (0.8-1.6) | 0.510 | 0.363 |
| TvE/Et | 5.4  (3.5-5.9) | 4.4  (2.6-4.7) | 0.182 | 4.3  (2.8-5.2) | 0.248 | 0.834 |  | 4.0  (3.8-5.7) | 4.8  (3.4-5.6) | 0.925 | 3.6  (3.0-4.7) | 0.594 | 0.155 |

Abbreviation: E, early transmitral flow velocity; A, late transmitral flow velocity; E/A, ratio of early to late transmitral flow velocity; Em, early diastolic mitral annular velocity; Am, late diastolic mitral annular velocity; E/Em, ratio of early transmitral flow velocity to diastolic mitral annular velocity; MPI, Myocardial Performance Index; TvE, early transtricuspid flow velocity; TvA, late transtricuspid flow velocity;TvE/A, ratio of early to late transtricuspid flow velocity; TvE/Et, ratio of early transtricuspid flow velocity to diastolic tricuspid annular velocity

**P*^a^< 0.05 compared with the corresponding parameters before and immediately after drainage. *P*^b^ < 0.05 compared with the corresponding parameters before and 24 hours after drainage. *P*^c^< 0.05 compared with the corresponding value immediately after drainageand the value 24 hours after drainage.

**Table S7.Correlation between the amount of drained PE and alterations of echocardio graphic measurements.**

| Variables | Effusion | | | |
| --- | --- | --- | --- | --- |
|  | First time Effusion  (r) | *P* value | 24h Effusion  (r) | *P* value |
| Difference of LVdVI |  |  |  |  |
| Immediately *vs*Before | 0.127 | 0.652 | - | - |
| 24 hours*vs* Before | - | - | 0.350 | 0.201 |
| Difference of LVsVI |  |  |  |  |
| Immediately *vs*Before | 0.025 | 0.929 | - | - |
| 24 hours*vs* Before | - | - | 0.465 | 0.080 |
| Difference of LAVI |  |  |  |  |
| Immediately *vs*Before | -0.150 | 0.593 | - | - |
| 24 hours*vs* Before | - | - | 0.278 | 0.316 |
| Difference of RV area |  |  |  |  |
| Immediately *vs*Before | 0.157 | 0.575 | - | - |
| 24 hours*vs* Before | - | - | 0.287 | 0.300 |
| Difference of RA area |  |  |  |  |
| Immediately *vs*Before | 0.025 | 0.929 | - | - |
| 24 hours*vs* Before | - | - | 0.501 | 0.057 |
| Difference of LVEF |  |  |  |  |
| Immediately *vs*Before | -0.033 | 0.907 | - | - |
| 24 hours*vs* Before | - | - | -0.323 | 0.240 |
| Difference of SV |  |  |  |  |
| Immediately *vs*Before | -0.101 | 0.720 | - | - |
| 24 hours*vs* Before | - | - | -0.326 | 0.236 |
| Difference of Sm |  |  |  |  |
| Immediately *vs*Before | -0.160 | 0.569 | - | - |
| 24 hours*vs* Before | - | - | -0.141 | 0.617 |
| Difference of GLS |  |  |  |  |
| Immediately *vs*Before | -0.089 | 0.753 | - | - |
| 24 hours*vs* Before | - | - | -0.262 | 0.346 |
| Difference of FAC |  |  |  |  |
| Immediately *vs*Before | 0.187 | 0.522 | - | - |
| 24 hours*vs* Before | - | - | -0.197 | 0.482 |
| Difference of TAPSE |  |  |  |  |
| Immediately *vs*Before | -0.175 | 0.532 | - | - |
| 24 hours*vs* Before | - | - | -0.254 | 0.361 |
| Difference of RVFWs |  |  |  |  |
| Immediately *vs*Before | -0.193 | 0.490 | - | - |
| 24 hours*vs* Before | - | - | -0.213 | 0.446 |
| Difference of IVRT |  |  |  |  |
| Immediately *vs*Before | -0.222 | 0.426 | - | - |
| 24 hours*vs* Before | - | - | -0.449 | 0.093 |
| Difference of E |  |  |  |  |
| Immediately *vs*Before | -0.399 | 0.141 | - | - |
| 24 hours*vs* Before | - | - | -0.411 | 0.128 |
| Difference of A |  |  |  |  |
| Immediately *vs*Before | 0.114 | 0.685 | - | - |
| 24 hours*vs* Before | - | - | -0.188 | 0.503 |
| Difference of E/A |  |  |  |  |
| Immediately *vs*Before | -0.211 | 0.450 | - | - |
| 24 hours*vs* Before | - | - | -0.280 | 0.313 |
| Difference of Em |  |  |  |  |
| Immediately *vs*Before | -0.267 | 0.336 | - | - |
| 24 hours*vs* Before | - | - | 0.025 | 0.929 |
| Difference of Am |  |  |  |  |
| Immediately *vs*Before | 0.084 | 0.765 | - | - |
| 24 hours*vs* Before | - | - | -0.338 | 0.217 |
| Difference of E/Em |  |  |  |  |
| Immediately *vs*Before | 0.189 | 0.499 | - | - |
| 24 hours*vs* Before | - | - | -0.109 | 0.711 |
| Difference of MPI |  |  |  |  |
| Immediately *vs*Before | -0.214 | 0.443 | - | - |
| 24 hours*vs* Before | - | - | -0.179 | 0.524 |
| Difference of TvE |  |  |  |  |
| Immediately *vs*Before | -0.345 | 0.227 | - | - |
| 24 hours*vs* Before | - | - | -0.047 | 0.874 |
| Difference of TvA |  |  |  |  |
| Immediately *vs*Before | 0.079 | 0.788 | - | - |
| 24 hours*vs* Before | - | - | -0.187 | 0.522 |
| Difference of TvE/A |  |  |  |  |
| Immediately *vs*Before | -0.376 | 0.185 | - | - |
| 24 hours*vs* Before | - | - | 0.164 | 0.574 |
| Difference of TvE/Et |  |  |  |  |
| Immediately *vs*Before | -0.321 | 0.263 | - | - |
| 24 hours*vs* Before | - | - | -0.047 | 0.874 |

Abbreviation: LVdVI, left ventricular end diastolic volume index; LVsVI, left ventricular end systolic volume index; LAVI, left atrial volume index; RV area, right ventricular area; RA area, right atrial area; LVEF, left ventricular ejection fraction; SV, stroke volume; Sm, systolic mitral annular velocity; GLS, global left ventricular strain; FAC, fractional area change; TAPSE, tricuspid annular plane systolic excursion; RVFWs, right ventricular free wall strain. E, early transmitralflow velocity; A, late transmitral flow velocity; E/A, ratio of early to late transmitral flow velocity; Em, early diastolic mitral annular velocity; Am, late diastolic mitral annular velocity; E/Em, ratio of early transmitral flow velocity to diastolic mitral annular velocity; MPI, myocardial performance index; TvE, early transtricuspid flow velocity; TvA, late transtricuspid flow velocity;TvE/A, ratio of early to late transtricuspid flow velocity; TvE/Et, ratio of early transtricuspid flow velocity to diastolic tricuspid annular velocity
